# Evaluating the risk of endometriosis based on patients’ self-assessment questionnaires

**DOI:** 10.1186/s12958-023-01156-9

**Published:** 2023-10-28

**Authors:** Krystian Zieliński, Dajana Drabczyk, Michał Kunicki, Damian Drzyzga, Anna Kloska, Jacek Rumiński

**Affiliations:** 1 INVICTA, Research and Development Center, Sopot, Poland; 2 Department of Biomedical Engineering, Faculty of Electronics, Telecommunications and Informatics, Gdańsk University of Technology, Gdańsk, Poland; 3 Department of Medical Biology and Genetics, Faculty of Biology, University of Gdańsk, Gdańsk, Poland

**Keywords:** Endometriosis, Questionnaire, Machine learning, Symptom-based prediction, Fertility

## Abstract

**Background:**

Endometriosis is a condition that significantly affects the quality of life of about 10 % of reproductive-aged women. It is characterized by the presence of tissue similar to the uterine lining (endometrium) outside the uterus, which can lead lead scarring, adhesions, pain, and fertility issues. While numerous factors associated with endometriosis are documented, a wide range of symptoms may still be undiscovered.

**Methods:**

In this study, we employed machine learning algorithms to predict endometriosis based on the patient symptoms extracted from 13,933 questionnaires. We compared the results of feature selection obtained from various algorithms (i.e., Boruta algorithm, Recursive Feature Selection) with experts’ decisions. As a benchmark model architecture, we utilized a LightGBM algorithm, along with Multivariate Imputation by Chained Equations (MICE) and k-nearest neighbors (KNN), for missing data imputation. Our primary objective was to assess the model’s performance and feature importance compared to existing studies.

**Results:**

We identified the top 20 predictors of endometriosis, uncovering previously overlooked features such as Cesarean section, ovarian cysts, and hernia. Notably, the model’s performance metrics were maximized when utilizing a combination of multiple feature selection methods. Specifically, the final model achieved an area under the receiver operator characteristic curve (AUC) of 0.85 on the training dataset and an AUC of 0.82 on the testing dataset.

**Conclusions:**

The application of machine learning in diagnosing endometriosis has the potential to significantly impact clinical practice, streamlining the diagnostic process and enhancing efficiency. Our questionnaire-based prediction approach empowers individuals with endometriosis to proactively identify potential symptoms, facilitating informed discussions with healthcare professionals about diagnosis and treatment options.

**Supplementary Information:**

The online version contains supplementary material available at 10.1186/s12958-023-01156-9.

## Background

Endometriosis is a medical condition characterized by the presence of the endometrial tissue (the mucous membrane of the uterus) outside the uterine cavity. It is estimated that about 1 in 10 women of reproductive age, or approximately 200 million women worldwide, may suffer from endometriosis, making its prevalence significant [1, 2]. The condition can persist for decades, starting as early as a woman’s first period and continuing beyond menopause [3]. Endometriosis is a heterogeneous disease with symptoms including menstrual pain, chronic pelvic pain, dyspareunia, and infertility. The severity of the disease is determined by the number and depth of endometrial lesions and often presents with comorbidities like migraines, irritable bowel syndrome, and chronic fatigue syndrome. The implications of endometriosis are multifaceted and can vary depending on individual experiences. It negatively impacts various aspects of quality of life and takes a toll on mental health [1]. Chronic pelvic pain can significantly disrupt daily activities, necessitating pain management strategies such as medications and lifestyle adjustments. Individuals may also experience stress, anxiety, depression, and frustration due to the unpredictability of symptoms and challenges in obtaining an accurate diagnosis and effective treatment [4].

Endometriosis is a common cause of infertility in women, often leading to adhesions and scar tissue that disrupt fallopian tube and ovarian function. This often necessitates fertility treatments like in vitro fertilization (IVF) for conception. Given a woman’s biologically limited reproductive timeframe, a quick diagnosis of endometriosis can significantly impact her chances of conceiving [5]. Currently, the typical diagnostic methods for endometriosis include patient interviews and ultrasound imaging (USG). However, USG may not always provide a complete and accurate diagnosis, especially in cases involving endometriosis in the uterus or ovaries. Often, confirmation of the diagnosis requires invasive and costly procedures such as laparoscopy or, in the case of extraperitoneal endometriosis, magnetic resonance imaging (MRI) [6]. Consequently, treatment decisions, including those related to IVF, often rely primarily on the patient’s medical history.

Numerous medical factors correlate with the likelihood of developing endometriosis, although a wide range of symptoms may still be undiscovered. For instance, early menarche [7–9] and shorter menstrual cycles [10] have been associated with a higher risk of endometriosis. Conversely, the use of oral contraceptive pills has been linked to a reduced risk [11]. Additionally, a consistent inverse correlation between body mass index (BMI) and endometriosis has been observed, possibly due to hormonal variations among women with different body weights [7]. However, the relationship between oral contraceptive pills and endometriosis risk is complex. Some studies indicate a decreased risk among current users but an increased risk among past users. Despite this, oral contraceptive pills are frequently prescribed to alleviate endometriosis-related pain, suggesting their effectiveness in suppressing symptoms of the condition [12].

Despite recent advancements in identifying risk factors for endometriosis, the field still faces the constraint of requiring surgical diagnosis to confirm the disease. During patient examinations, doctors gather a wealth of information, some of which may lead to conflicting conclusions about an endometriosis diagnosis. Consequently, there is a pressing need for a unified method that accounts for valid factors and calculates the likelihood of having endometriosis.

The scientific community has demonstrated a growing interest in developing methods for diagnosing endometriosis [13]. There is an increasing reliance on modern statistical methods, such as machine learning, to enhance this process. The development of a predictive model for endometriosis diagnosis could enable healthcare providers to identify the condition earlier and more accurately, thereby improving patient outcomes and optimizing the use of healthcare resources. However, the development of such a predictive tool presents several challenges, including the availability of suitable training data.

In this study, we administered a custom questionnaire, meticulously crafted by experienced reproductive medicine specialists, to patients before their initial visit to an infertility treatment clinic. We analyzed the data collected from these questionnaires to identify the most crucial features for predicting endometriosis. Both gynecologists and machine learning techniques were employed to identify these key features. The primary goal of our research was twofold: first, to assess the feasibility of training a predictive model for endometriosis, and second, to compare the significance of these features to those identified in existing studies. Finally, we provided clinicians with a model that predicts the likelihood of endometriosis, complete with an explanation.

## Methods

### Ethics and consent to participate

The study is a retrospective study based on anonymized datasets and therefore does not require the consent of the ethical committee or the patients.

### Dataset

The study utilized retrospective data from the Invicta database, which maintains comprehensive patient records. The study group comprised patients diagnosed with endometriosis by medical professionals at Invicta clinics, in accordance with the standards set by the European Society of Human Reproduction and Embryology (ESHRE). The control group included both infertility patients not diagnosed with endometriosis and egg donors without known fertility issues. Relevant attributes considered potentially valuable predictors were extracted and merged into a single dataset. The dataset was limited to self-assessment questionnaire responses collected from June 2018 to August 2022 and included attributes characterizing patients, visits, questionnaire questions, and responses. A total of 13,933 questionnaires were processed. The questionnaire contained 272 patient-related questions, 134 of which were selected for further analysis; the rejected questions pertained to information such as e.g., eye color or hair shape.

To process the data, questionnaire answers were categorized into groups for which processing functions were developed. Each function converted the questionnaire answers into a table where columns represented questions and answers. For questions with *n* possible answer options, *n* columns were generated. The answer formats in the questionnaire data included single-select, binary, multi-select, date, numeric, and mixed. Quantitative variables were bounded by minimum and maximum thresholds determined by experts to eliminate extreme values. Categorical variables were transformed into a dichotomous form, and ordinal coding was used for ordinal variables. Attributes conveying equivalent information from different questionnaire questions or answers were merged. The resulting data frame consisted of 204 columns and 11,819 rows, with the visit ID serving as the index. Note that the number of columns exceeds the number of questions because some questions were of the multi-select type; thus, the number of features increased after one-hot encoding.

The target feature in this study was the diagnosis of endometriosis, obtained either from the patient’s medical history or the qualifying questionnaire routinely administered at the outset of assisted reproductive technology procedures at Invicta clinics. Using regular expression matching operations, we scoured the database for instances of endometriosis diagnosis and subsequently integrated these findings with the responses from the qualification questionnaire. This resulted in a target feature consisting of 910 labels denoting an endometriosis diagnosis. Comprehensive statistics characterizing the study population are presented in Table 1.

**Table 1:** Basic statistics describing the study population. For features with binary responses, 0 indicates a negative answer, while 1 indicates an affirmative response to a questionnaire question

Feature	% of affirmative or Mean	Data available	Min	Max
Patient age (years)	34.69	11819	20	55
Appendectomy	21.27%	4433	0	1
Caesarean section	4.85%	4433	0	1
Drainage fallopian tubes	9.42%	11363	0	1
Infertility diagnosis	20.39%	11446	0	1
Hernia	3.16%	4433	0	1
Pelvic inflammatory disease (PID)	1.42%	11456	0	1
Few months of trying to get pregnant	29.87%	11819	0	1
Less than 3 years of trying to get pregnant	63.39%	11819	0	1
Over 3 years of trying to get pregnant	3.87%	11819	0	1
Ovarian cysts	11.69%	11287	0	1
No periPelvic pain	11.52%	11819	0	1
Moderately severe periPelvic pain	45.84%	11819	0	1
Very strong periPelvic pain	16.45%	11819	0	1
Recurrent vaginitis	13.53%	11456	0	1
Reduction of sex drive	8.10%	11150	0	1
Removal of ovarian cysts	3.54%	11284	0	1
Average menstrual cycle length (days)	28.66	10659	18	40
Body mass index (BMI)	23.50	10874	15.9	46.9
Number of pregnancies	0.62	4321	0	7
Number of miscarriages	1.11	4321	0	10
Endometriosis	10.46%	11819	0	1

### Feature selection

Feature selection is a critical step in machine learning because it enhances model performance, reduces computational complexity during training, and improves result interpretability. The quality of the features used to train a model can significantly affect its performance. Choosing the most informative features not only improves the model’s accuracy but also minimizes overfitting and prevents the model from learning irrelevant or noisy patterns in the data. Moreover, it considerably reduces the computational complexity of the training process, thereby expediting training faster, especially for large datasets with numerous features [14].

**Fig. 1:**
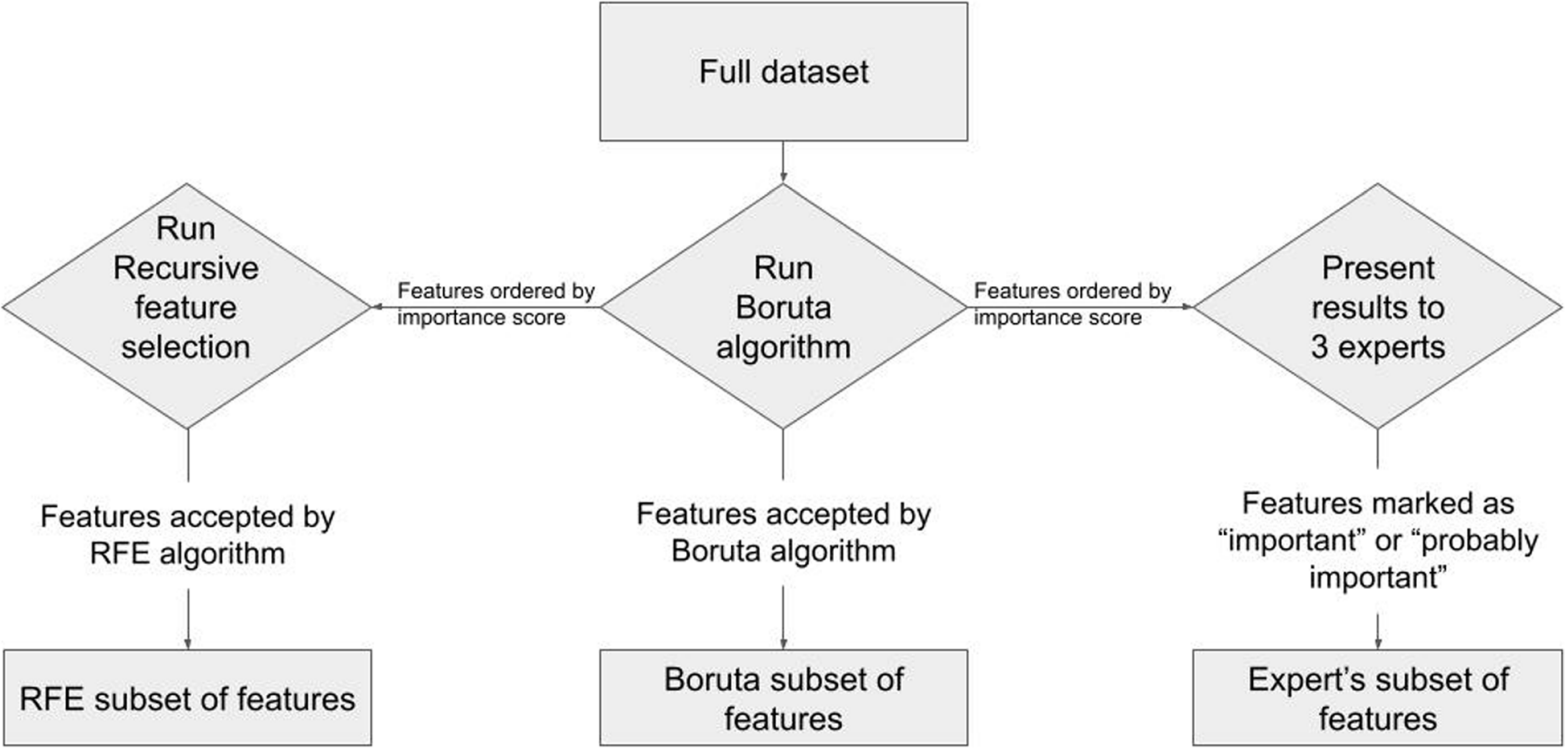
Diagram of feature selection process incorporated in the study

In our study, we conducted experiments using the complete dataset and applied three different feature selection methods, complemented by experts’ decisions and statistical analysis. A key strategy was multistage feature selection to ensure that the trained models were not overfitting due to the inclusion of non-informative features. We also took additional measures, including specific hyperparameter settings and 5-fold cross-validation, and performed 25 replications of the process using different seeds to split the data into 5 folds, in order to evaluate overfitting. Furthermore, the features selected via statistical methods enabled us to compare existing knowledge with potential endometriosis predictors that might not have been previously identified. The process of feature selection is visualized in Fig. 1.

The first step in feature selection involved the application of the Boruta algorithm [15–17] to the dataset. Originally based on the Random Forest machine learning model, the algorithm compares the importance of each feature in the original dataset with the importance of that same feature when randomly shuffled. To begin, the original dataset was duplicated and appended with randomly shuffled copies of each feature. A Random Forest model was then trained on this augmented dataset. This model assigned an importance score to each feature based on how well it separated the target variable. The algorithm compared the importance score of each feature with the scores of its shuffled counterparts. If a feature’s importance score significantly exceeded that of its shuffled copies, it was deemed significant. Features not classified as significant were removed, and the process was repeated until only significant features remained.

The Boruta algorithm is particularly useful for identifying relevant features in high-dimensional datasets, where the number of features greatly exceeds the number of observations. It can identify complex feature interactions and select features that are relevant for predicting the target variable. Furthermore, the Boruta algorithm is resistant to noisy or redundant features, making it a robust method for feature selection.

In the next step, all original variables, along with their Boruta rankings as additional information, were presented to three experts. Their task was to evaluate the relevance of these attributes for predicting endometriosis based on their expertise. The experts used a fixed mapping system to label each variable. Variables were marked with ‘-1’ when experts were certain that they did not correlate with endometriosis. Attributes marked with ‘0’ were considered to possibly affect prediction, while those marked with ‘1’ were highly recommended for prediction. A set of variables, consisting of those marked with ‘0’ and ‘1’, was then used to determine feature importance. Additionally, this expert classification allowed us to compare attributes identified by experts and those that were top-ranked by machine-learning algorithms.

Another technique used in the study to determine the most important factors for endometriosis was Recursive Feature Selection (RFE) [18, 19]. The RFE algorithm recursively eliminates features from the dataset and ranks the remaining ones based on their importance. The rationale behind RFE is that by removing the least important features, the model’s performance will either remain unchanged or improve, as it will focus on the most significant features.

As differences between single model runs may vary, the process of RFE in the study can be described in the following steps:

1. It first trains a model on the entire set of features and calculates the importance of each one.
2. Features are ordered according to their Boruta score. The least important feature as identified by the Boruta algorithm, is removed. A model is then trained 25 times using different initial random states.
3. At each step, the model’s performance is evaluated using the area under the receiver operator characteristic curve (AUC-ROC score).
4. Using statistical test (Kolmogorov-Smirnov) [20], the distribution of AUC-ROC scores for the model trained on the current subset is compared with that of the model trained in the previous step
5. If the test shows that there are no statistical differences between model scores, a feature is removed from the training set.
6. The algorithm stops when all of the features have been tested.

### Model

As a benchmark model architecture, a gradient boosting technique [21] was applied. We selected the LightGBM [22] implementation of the algorithm because it is characterized by high performance and has built-in capabilities for handling missing data. In the context of medical questionnaires, handling missing data is a major concern. LightGBM can manage missing values by treating them as separate categorical values. When building decision trees, the algorithm creates a separate branch for missing values and assigns weights to them based on their relative importance to the target variable. Training the algorithm is an iterative process that begins by initializing the model with a single decision tree that has a single root node containing the mean value of the target variable. The algorithm then iteratively trains a series of decision trees. Each new tree is trained to correct the errors made by its predecessors. During each iteration, the algorithm calculates the gradients and Hessians of the loss function with respect to the predicted values and updates the model accordingly. To determine splits in each tree, the algorithm searches for the best-split point that maximizes the reduction in the loss function.

To prevent overfitting, various regularization methods [23] were incorporated. These include feature bagging, data bagging, l1 and l2 regularization on weights. To assure weak learners, the maximum depth and the maximum number of leaves for each tree were set.

The hyperparameters of the model were determined using random grid hyperparameter optimization [24]. This technique allows for the identification of the best hyperparameters for a machine-learning model by randomly sampling values from a predefined range of hyperparameters. This method was used in conjunction with cross-validation to find the hyperparameters that produced the highest cross-validation score. Random grid samples are combinations of parameters drawn from a defined parameter space. In each iteration, the selected hyperparameters were used to train and evaluate the model using cross-validation. The performance of the model was evaluated based on the AUC score.

The random grid search method can efficiently explore the hyperparameter space and find a suitable set of hyperparameters for the model without the need for an exhaustive search across the entire space. This approach is particularly useful for high-dimensional search spaces where an exhaustive search is computationally infeasible.

**Figure:**
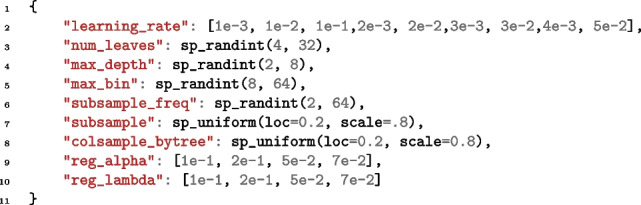
**Listing 1** Hyperparameter space used in the study

### Missing data imputation

To test other types of model architectures, missing values in the dataset had to be imputed. The task was exceptionally complex due to various potential reasons for missing values, e.g., patients may have opted not to disclose certain information for personal reasons, some questions were dependent on other answers (e.g., a patient who never took a particular medication wouldn’t answer dosage-related questions), or certain questions were not available in specific versions of the questionnaire. To handle this, we compared the results of imputation methods using the mean and median with iterative methods such as the Multivariate Imputation by Chained Equations (MICE) [25] and k-nearest neighbors (KNN) algorithm [26].

For the MICE algorithm, we used the IterativeImputer implementation available in sklearn [27]. This iterative method imputes missing values in a dataset by modeling each feature with missing values as a function of the other features in that dataset. The algorithm starts by initializing the missing values with an initial value, such as the mean or median of the feature. It then iteratively imputes the missing values by modeling each feature with missing values as a function of the other features. The imputation is done round-robin, where each feature is imputed in turn. For each feature with missing values, a regression model is trained using the other features in the dataset as predictors. The model is used to predict the missing values for that feature. While in the original MICE paper, the algorithm used linear regression to determine missing values, IterativeImputer allows the use of other architectures. In this study, we opted for the Bayesian Ridge algorithm [28] due to its reduced computational time. The algorithm repeats the feature model training and imputation steps for a predefined number of iterations or until the imputed values converge.

The KNN algorithm can also be used for missing data imputation. It identifies the nearest neighbors for each observation with missing values and uses the feature values of those neighbors to determine the missing value. The algorithm identifies the k-nearest neighbors for each observation based on a selected distance metric. Next, it imputes the missing value by taking the average (for continuous variables) or the mode (for categorical variables) of the values of k-nearest neighbors. This process is repeated for each missing value in the dataset. One of the advantages of using the KNN algorithm for missing data imputation is its ability to handle both categorical and continuous variables. Additionally, KNN works well for datasets with nonlinear relationships between features.

### Software

This study was conducted on the Ubuntu 20.04.5 LTS version of the operating system, with an 11th Gen Intel®Core™ i7-11800H @ 2.30GHz $\times$ 16 processor and a GPU GeForce RTX 3050 Ti Mobile. Python version 2.7.18 was used for the study.

## Results

### Results of feature selection

Out of 258 initial features, 20 were selected by the Boruta algorithm, 67were chosen by experts, and 165 were picked using the RFE algorithm. Interestingly, three features selected by Boruta-namely frequent urination, headaches, and reduction of sex drive-were not chosen by RFE. Additionally, experts did not select 12 features that were picked using the Boruta algorithm and 13 features considered important by the experts were not selected by any of the feature selection methods; these included ovarian cysts, hysteroscopy, appendectomy, hernia, fallopian tube drainage, disturbing symptoms related to the urogenital system, feeling overall healthy, spooning, cytomegaly, Caesarean section, and tonsils. A full list of selected features by each method is available at Additional file 1.

To optimize the parameters of the LightGBM classification model, a random grid search was run for each subset of features. The algorithm generated 1,000 different versions of the model. The best parameters were then chosen based on a 3-fold cross validation AUC score.

**Table 2:** Model hyperparameters selected for models trained on a subset of features selected by a given method

Method	colsample bytree	learning rate	max bin	max depth	num leaves	reg alpha	reg lambda	subsample	subsample freq
Boruta	0.50	0.10	39	3	31	0.20	0.20	0.30	51
Experts’ decisions	0.29	0.03	46	6	20	0.05	0.07	0.76	48
Recursive selection	0.28	0.05	53	5	9	0.10	0.07	0.75	47

Using the selected hyperparameters shown in Table 2, models were trained using 5-fold cross-validation. The performance metrics are shown in Table 3. To ensure the robustness of the results, experiments were repeated 25 times, each with a different random seed for cross-validation split.

**Table 3:** Metrics obtained by models trained on a subset of features selected by a given method

Method	Precision	Recall	Specificity	Accuracy	F1 weighted	Test AUC	Train AUC	Matthew’s coefficient
Boruta	0.26	0.69	0.77	0.76	0.80	0.80	0.81	0.32
Experts’ decisions	0.23	0.69	0.73	0.72	0.77	0.78	0.85	0.28
Recursive selection	0.25	0.74	0.73	0.73	0.78	0.81	0.85	0.31

Based on the model’s results, the best error metrics were obtained using the subset of features selected by RFE, with an average AUC above 0.81. In contrast, the model trained on features selected by experts achieved the worst error metrics, with an AUC below 0.78. These error metrics are shown in Table  3. Although the Boruta algorithm selected only 20 important columns, the model trained on this subset achieved an AUC of 0.8, comparable to that obtained for recursive feature selection. The model trained on features selected by experts had the lowest evaluation metrics. The model trained with RFE-selected features had the highest recall, while the model trained with Boruta-selected features achieved the highest Matthew’s coefficient. Additionally, a comparison of the AUC metrics obtained for the train and test subsets shows that the model trained with Boruta-selected columns is the most robust. The difference between the AUCs calculated on the train and test subsets was only 0.01. In contrast, the overfit was 0.07 and 0.04 for models based on expert-selected and RFE-selected features, respectively. While higher, these levels of overfit are still acceptable.

Using different random seeds did not impact the AUC metrics for any of these models; the difference between the first and third quantiles of the results was below 0.005, confirming the method’s stability.

### Imputation techniques and final model

**Fig. 2:**
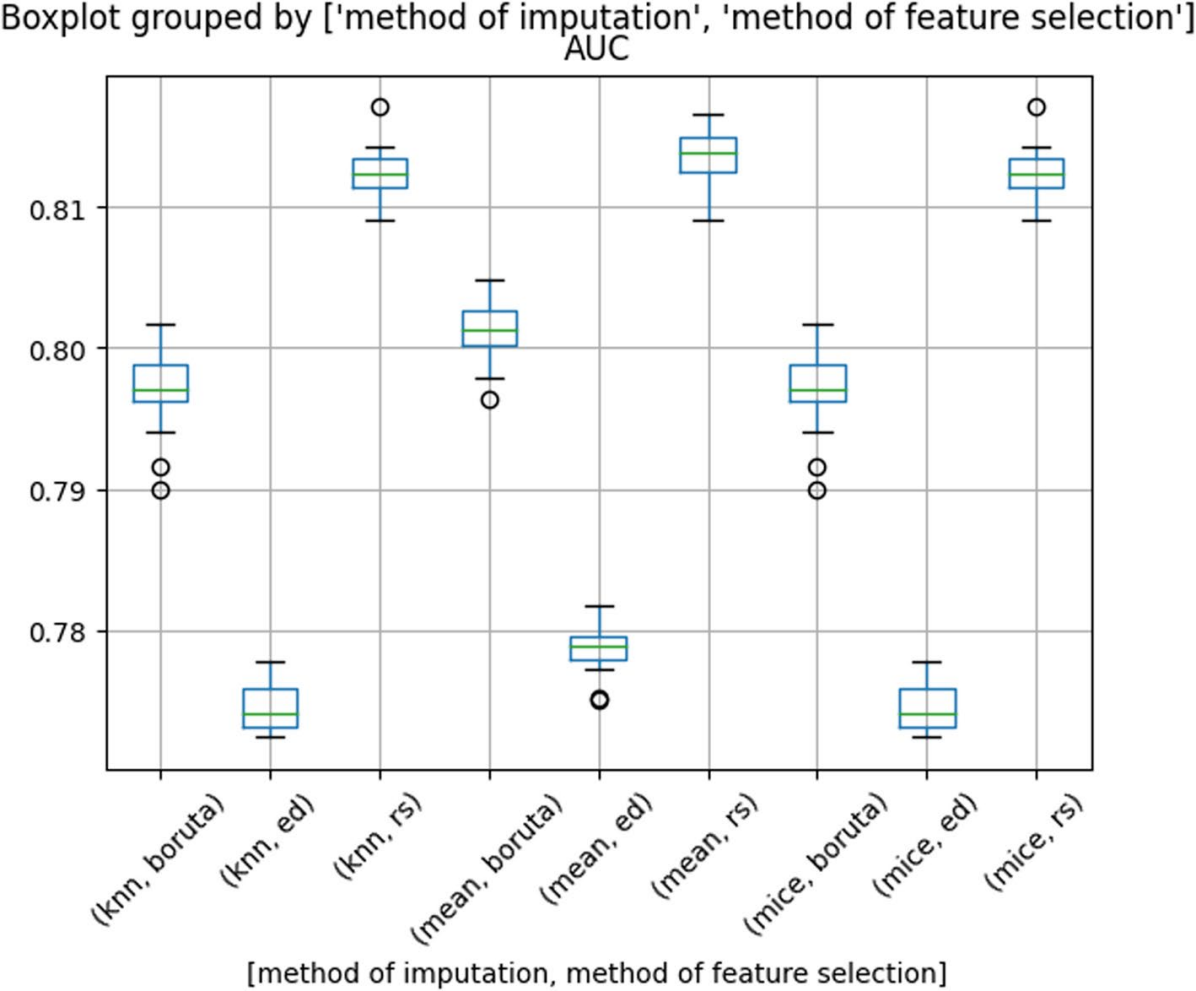
Boxplot of the area under the curve (AUC) values for different cross-validation splits after imputation with different methods. The difference between Q1 and Q3 for most cases is less than 0.01; therefore, the model’s training process can be evaluated as stable. There is a much bigger difference between methods of feature selection than between imputation techniques. In each case, the recursive selection was superior compared to Boruta. Experts’ decisions appear to be the least effective method of feature selection

The study demonstrated that the choice of feature subsets significantly affects the performance of the models. To fully understand the impact that different imputation techniques might have on the modeling, experiments were conducted separately for each column subset identified in previous steps. Three different imputation methods were used: KNN, mean, and MICE, and each was applied to all feature subsets. The results, presented in Fig. 2, reveal very low variability in the models’ performance depending on the imputation method used. Most differences in performance occur due to varying feature sets. Using KNN and MICE resulted in a slight decrease in AUC for feature subsets selected by the Boruta and expert decision methods, as compared to models that did not employ any imputation. Imputing average values into each feature did not impact the models performance. Overall, the findings suggest that imputation does not always improve model performance. However, using these techniques allows researchers take advantage of different models that are otherwise unable to handle missing values.

Since the optimal column subset selection was disputed, an additional model was trained to supplement the initial results. The final feature subset included all features selected by the Boruta algorithm, as well as the top 20 features ranked by SHAP values in other models but not included in the Boruta subset of features. Nine of the newly added features had been selected by experts. These were: BMI, patient age, longest menstrual cycle length, shortest menstrual cycle length, average menstrual cycle length, number of pregnancies, number of miscarriages, length of trying to get pregnant, oral contraceptive pills. Additionally, featrues that were highly ranked in the model trained on the RFE feature subset were included, i.e., Pelvic inflammatory disease (PID) and First menarche. The new feature subset comprised 30 columns.

For this selected subset of features, the following GBM parameters were chosen:

{ "colsample_bytree": 0.49, "learning_rate": 0.03, "max_bin": 41, "max_depth": 4, "num_leaves": 25, "reg_alpha": 0.2, "reg_lambda": 0.2, "subsample": 0.83, "subsample_freq": 42 }.

**Fig. 3:**
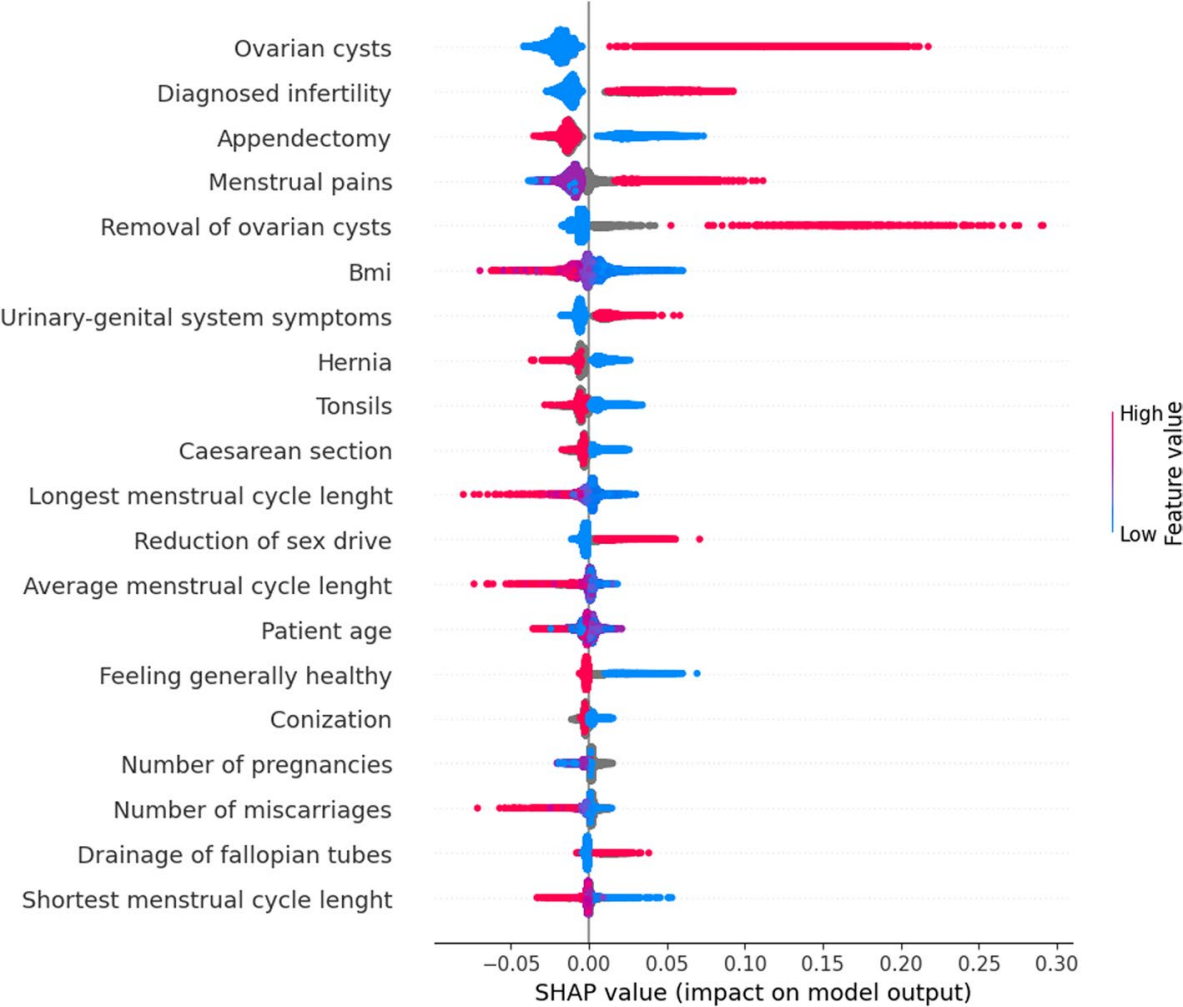
The 20 most important features are sorted by the magnitude of the SHapley Additive exPlanations (SHAP) values. Plots display the SHAP values for each feature of a given observation in a horizontal orientation. Each dot on the plot represents an individual observation and the position of the dot on the x-axis represents the magnitude of the SHAP value. The color of the dot represents the value of the corresponding feature for that observation, with red indicating high feature values and blue indicating low feature values

Models were trained 25 times using different seeds in 5-fold cross-validation to ensure the stability of the results. For each run and each split, evaluation metrics were calculated for both the training and testing subsets. This resulted in 5 train evaluation metrics and 5 test evaluation metrics for each of the 25 runs. Next, the error metrics from each run were averaged. The selected model was explained using Shapley additive explanations (SHAP) values to assess the impact of each feature on the model’s output (Fig. 3). To further analyze the impact of the features, a correlation matrix was calculated. For features on interval and ratio scales, Spearman’s correlation coefficient was used, while Matthew’s correlation coefficient was used for binary features (Supplementary Table 1). The highest correlation was noticed for frequent urination, reduction of sex drive, and urinary-genital system symptoms. Among features on the ratio scale, the strongest correlation was found between the average menstrual cycle length and the shortest/longest menstrual cycle length.

**Table 4:** Metrics obtained by the final model

Subset	Precision	Recall	Specificity	Accuracy	F1 weighted	AUC	Matthew’s coefficient
Train	0.29	0.76	0.78	0.78	0.82	0.85	0.37
Test	0.26	0.73	0.76	0.76	0.80	0.82	0.33

The model achieved the highest performance metrics among all experiments (Table  4) demonstrating the advantages of using a combination of multiple methods for feature selection. Features with the highest positive correlation with endometriosis included ovarian cysts, diagnosed infertility, high pelvic pain, disturbing symptoms related to the urogenital system, and reduction of the sex drive. Features that negatively correlated with endometriosis included appendectomy, high BMI, tonsils, hernia, Caesarean section, and shorter menstrual cycle. The impact of patient age on endometriosis diagnosis appears to be non-monotonic and may depend on other features.

## Discussion

In this study, we demonstrated that a preliminary diagnosis of endometriosis can be made based on a simple questionnaire containing specific questions. We identified the top 20 predictors of endometriosis, including some previously overlooked features like Cesarean section, ovarian cysts, and hernias. We also confirmed a strong correlation between menstrual pains and the likelihood of having endometriosis. Additionally, our approach of multi-step feature selection improved the models robustness.

The delayed diagnosis of endometriosis underscores the need for a simple and reliable screening tool to identify women at higher risk. Previous studies have used questionnaires completed by patients as initial screening tools for endometriosis [29]. These questionnaires often included detailed inquiries about factors such as the age of menarche, cycle duration, dysmenorrhea, pain descriptors, dyschezia, urinary symptoms, ovarian cysts, diagnosed infertility, appendectomy, and pelvic pain diarrhea [30–34]. Although several studies have attempted to develop mathematical models based on self-administered or preoperative questionnaires to predict endometriosis [35, 36], some of them were complex or required additional diagnostic parameters (e.g., ultrasound and pelvic examination), making them impractical for patient self-completion. Additionally, certain measures were limited to specific populations (e.g., women with site-specific endometriosis or deep-infiltrating endometriosis) or had lower accuracy rates for early-stage endometriosis. In our study, similar features were confirmed to have high predictive value. Furthermore, our findings revealed additional symptoms such as Cesarean section, ovarian cysts, and hernia, which had not been previously considered predictors.

The primary challenge for applying machine learning algorithms in healthcare is the need for large amounts of high-quality data. For a relatively rare condition such as endometriosis, a prediction model relies on accurate and representative patient data. A prediction model must be rigorously tested and validated in a variety of patient populations to ensure that it is accurate and reliable. Obtaining this requires access to large and diverse patient populations. Datasets from questionnaires can be challenging for several reasons - missing data, inconsistencies, and quality issues. Missing data can be caused by questionnaire non-responses or invalid responses. Questionnaires may also have inconsistencies in the data, such as duplicate responses or responses that do not match the question, while quality issues can arise from questionnaire design, respondent bias, or other factors. To ensure the reliability and validity of the data, it is essential to identify and correct these issues. Additionally, the whole process of training the model using multiple feature selection methods and hyperparameter optimization can have a high time complexity.

It is crucial to emphasize endometriosis’s diverse trajectory throughout a woman’s life. This diversity is reflected in the wide range of symptoms, disease progression, and treatment responses that women with endometriosis experience [37]. To effectively track the complex longitudinal changes in endometriosis features, it is important to leverage machine learning models tailored for longitudinal data, such as recurrent neural networks (RNNs) or specific survival analysis models. These models can capture the dynamic relationships between features over time and identify patterns that may be difficult to detect using traditional statistical methods. In the feature studies by leveraging machine learning models, researchers can gain a deeper understanding of the inherently diverse trajectory of endometriosis and develop more personalized and effective treatment strategies.

The results of our study were compared with a similar study predicting endometriosis based on data from the UK Biobank [38]. It should be noted that this study included a more comprehensive range of patient medical and genetic data; therefore, comparing the two studies at the feature level could be biased. Of the top 20 features identified in the study based on the UK Biobank data, only seven were also included in the top 20 of the features of both studies (Table  5). In our dataset, eight features that were indicated as important in other studies were not available for the current study. Additionally, five features were excluded during the feature selection process.

**Table 5:** Feature comparison between models based on data from the UK Biobank and INVICTA questionaries. It should be noted that certain features are absent in the INVICTA dataset due to their non-inclusion in the patient questionnaire, either in the specified format or any other form that would enable a direct match with corresponding features in the UK Biobank data

Feature	UK Biobank	INVICTA
length of menstrual cycle	0	12
age at first live birth	1	Not available in the data
n92 - excessive, frequent and irregular menstruation	2	Not selected to the modelling
number of live births	3	16
n83 - noninflammatory disorders of ovary, fallopian tube and broad ligament	4	Not available in the data
stomach/abdominal pain for 3+ months	5	Not selected to the modelling
source of report of k58 (irritable bowel syndrome)	6	Not available in the data
UK Biobank assessment centre	7	Not available in the data
pelvic inflammatory first	8	<20
n94 - pain and other conditions associated with female genital organs and menstrual cycle	9	4
degree bothered by menstrual cramps	10	4
year of birth	11	15
estrogen exposure	12	Not available in the data
n97 - female infertility	13	3
n81 - female genital prolapse	14	Not available in the data
irregular cycle	15	Not selected to the modelling
n84 - polyp of female genital tract	16	Not selected to the modelling
n73 - other female pelvic inflammatory diseases	17	<20
o70 - perineal laceration during delivery	18	Not available in the data
n85 - other noninflammatory disorders of uterus except cervix	19	Not available in the data
body mass index (BMI)	20	6

Compared to the study [38], the length of the menstrual cycle, the number of live births, and pelvic inflammatory disease were noticeably less important for prediction in our study. On the other hand, diagnosed infertility had a higher impact on the model’s output. The study based on the UK Biobank data showed a low impact of the BMI on the prediction, whereas the model trained on Invicta data this feature was ranked higher. Neither study identified The age of menarche as a strong predictor of endometriosis. When comparing AUCs, the model trained with INVICTA data performaned slightly better, with an AUC of 0.82 against the 0.79 for the UK Biobank data.

**Table 6:** Metrics obtained by the final model

Study dataset	Precision	Recall	Accuracy	F1	AUC	% of endometriosis
UK Biobank	0.50	0.30	0.92	0.37	0.78	0.04
Invicta	0.26	0.73	0.76	0.38	0.82	0.10

Comparison of other metrics, as shown in Table 6, highlights the different approaches in both studies for selecting the optimal threshold between labels “sick” and “not sick”. In the current study, the optimal threshold was selected as the point closest to (0,1) on the ROC curve. Higher recall scores mean fewer false negative predictions, i.e., fewer sick patients misclassified as patients without endometriosis. If the model is to be used as a screening tool, this approach would be beneficial for patients with the lowest probabilities of having endometriosis, as additional medical exams would not be necessary. Additionally, patients with a higher probability of having endometriosis could undergo further examination to confirm or exclude the diagnosis. Based on discussions with practitioners, it is advised to provide both the probability of diagnosis and the percentage of patients who had the same or lower probability of endometriosis. This generally makes the interpretation of scores easier for gynecologists.

In the fields of medical research and healthcare, the availability of diverse datasets from various medical centers offers valuable opportunities for developing predictive models and extracting meaningful insights. However, comparing the results of modeling across these datasets presents several challenges resulting from variations in data collection protocols, patient demographics, and healthcare practices. Such differences can introduce inconsistencies in model performance and hinder the generalizability of findings. Therefore comparing the results of the two studies can result in misleading conclusions.

Our study has several limitations. The first is the use of a non-validated questionnaire; however, its reliability and validity as a data collection tool is guaranteed by its long-term use in the clinical setting. Over 15 years of use and feedback from gynecologists working with it suggest that the tool is robust and has been refined over time for clinical relevance. The questionnaire covers a wide range of topics relevant to infertility, including phenotypic features, treatment history, general health (including menstrual cycle, drugs, and lifestyle), and genetic factors, and it includes a statement for patients to confirm the truthfulness of their answers adding a level of accountability to the data. It has to be filled out using a patient’s online account and the system has built-in forms of data validation. The questionnaire is integrated into the clinics’ hospital information system, making it accessible to the treating physician and ensuring it is part of the patient’s medical record.

As each medical center may follow its procedures for data gathering, including variations in data formats, missing data handling, and feature engineering techniques the differences resulting from those aspects can introduce bias and make direct comparisons challenging. To address this issue, it is crucial to establish standardized protocols for data collection across medical centers. Standardization should include the selection and definition of variables, data preprocessing techniques, and handling of missing data. By adopting standardized procedures, the comparability of datasets can be improved, enabling more meaningful comparisons of modeling results. Additionally, this would enable researchers to use transfer learning in the larger spectrum of domains.

Another limitation of our study is its reliance on retrospective data and diagnoses provided by medical professionals. Although these diagnoses adhere to the guidelines of the European Society of Human Reproduction and Embryology (ESHRE), it should be noted that not all might have been based on histological findings, traditionally considered the gold standard for diagnosing endometriosis; unfortunately, these data were not available in our dataset. However, current ESHRE guidelines indicate that advances in imaging technologies have necessitated a reevaluation of this gold standard [39, 40]. In this light, it is possible that not all of those diagnoses were based on histological findings as it is a standard that requires a serious medical intervention whose risks often outweigh the risks of starting treatment, and hence in a clinical setting it is not always employed. Due to these limitations of our retrospective data, we were unable to categorize endometriosis by type and stage in the study group to explore the sensitivity of the final model in response to endometriosis type. However, we acknowledge that this would be a valuable direction for future research.

The use of convenience-based data integration for parameter selection excluding some traits such as eye color can also be recognized as a limitation of the study. Recent findings found an association between certain pigmentation traits, such as green eyes and blonde/light brown hair [41] or blue eyes [42] and endometriosis risk potentially hinting at genetic or environmental factors contributing to endometriosis development. Exploring these traits may also have implications for understanding the disease’s pathogenesis.

A limitation also lies in the use of experts’ knowledge to identify a list of features important for predicting endometriosis. Such data should be approached with caution. Experts’ decisions can be subjective and may vary among individuals. Errors in judgment or personal biases can affect their assessments.

## Conclusions

The use of patient-completed questionnaires as screening tools for endometriosis holds the potential to identify individuals at increased risk. Patient-based screening tools combined with ML can lead to empowering patients to self-identify symptoms and consult their symptoms with healthcare professionals. Our study demonstrates that research is still required to determine clinical factors associated with endometriosis, not only to investigate the common, medically-confirmed factors but also to pinpoint new ones. Ongoing validation and research are essential to establish an effective and accurate screening tool for endometriosis.

### Supplementary Information

**Additional file 1.** Supporting information.

**Additional file 2.** Supplementary Table 1. Correlation matrix.

## Data Availability

INVICTA Fertility Clinics do not allow public disclosure of patient data used in this study. In case of additional questions, please contact the authors or INVICTA Research and Development Center(cbr@invicta.pl). The source code is available at the GitHub repository (https://github.com/CBR-Invicta/Endometriosis)-notebooks contain results and plots generated using the dataset.

## Abbreviations

AUC-ROC: Area under the receiver operator characteristic curve
BMI: Body mass index
IVF: In vitro fertilization
RFE: Recursive Feature Selection
KNN: K-nearest neighbors
MICE: Multivariate imputation by chained equations
MRI: Magnetic resonance imaging
PCOS: Polycystic ovary syndrome
PID: Pelvic inflammatory disease
SHAP: SHapley Additive exPlanations
USG: Ultrasound imaging
