# Supplementary material for: Evaluating the risk of endometriosis based on patients’ self-assessment questionnaires
Source: Reprod Biol Endocrinol. 2023 Oct 28;21:102. doi: 10.1186/s12958-023-01156-9 (PMC10612251; doi:10.1186/s12958-023-01156-9)
Supplement: Supplementary file 1 — Additional file 1. Supporting information. [file 12958_2023_1156_MOESM1_ESM.pdf]

Supporting information

S1 Appendix. Features selected by each method.

Boruta: [Urinary-genital system symptoms, Hysteroscopy, Recurrent vaginitis, Tonsils, Myomas, Appendectomy, Diagnosed infertility, Pelvic pain, Cytomegaly, Removal of ovarian cysts, Frequent urination, Reduction of sex drive, Cesarean section, Ovarian cysts, Headaches, Cervical conisation, Drainage of fallopian tubes, Spooning, Hernia, Feeling generally healthy]

Experts' decisions: [Years of trying to get pregnant, Menstrual symptoms, Bleeding after intercourse, Recurrent vaginitis, Urinary incontinence, Patient age, Weakness, Number of pregnancies, Longest menstrual cycle length, Urethritis, Myomas, Low blood pressure, Anemia, Diagnosed infertility, Number of biological children with current partner, Unexplained vaginal bleeding, Pains on walking, Weak appetite, Mental issues, Abundance of menstruation, More than two miscarriages, No anticonception, Nausea, Number of biological children, Heat intolerance, Sinusitis, Number of miscarriages, The average duration of menstruation, Headaches, Pylyps, Shortest menstrual cycle length, Dysuria, Persistent rash, Mood changes, Vascular thrombosis, Amenorrhoea, Cholecystolithiasis, Intermenstrual spotting, Sepsis, Recurrent kidney infections, Enlarged and sore breasts, Breast lumps, Rheumatic diseases, Pelvic pain, Irregular periods, The maximum difference between the shortest and longest periods, Frequency of sexual intercourse, Ectopic pregnancy, High blood pressure, Number of miscarriages with current partner, Frequent urination, Reduction of sex drive, Pelvic inflammatory disease (PID), Average menstrual cycle length, Stool problems, Oral contraceptive pills, Rh-, First menarche, Sleep disorders, Age at menarche, PCOS (polycystic ovary syndrome), BMI (body mass index), Recurrent miscarriages, Cervical conisation, Spit out of breast nipple, Intrauterine injection, Dizziness]

Recursive Feature Selection:

[Condom, Fever, Vision problems, Lymph node enlargement, Kidney stones, Medroxyprogesttablets medicine, Patient age, Premature extinction of ovarian function, Epilepsy, Clomifen tablets, Bromocriptine medicine, Excessive sweating, Weight increase of 5 kg, Myomas, Tuberculosis, Appendectomy, Gastric ulcer of the stomach, Removal of ovarian cysts, Cesarean section, Willow indifferent, Moles change colors, Herpes simplex, Mental issues, Liver enlargement, Disturbing symptoms related to the skeleton-muscular-articular system, Mumps, Blood group type 0, Folic acid medicine, Unexplained weight loss, Breast glands related symptoms, Strange stains in mouth, PGT-A (Preimplantation Genetic Testing for Aneuploidy), Sinusitis, Persistent sore throat, Hearth diseases, Intolerance to food, Spermicide, Muscular tremor, Don't know the blood Rh factor, IUI (intrauterine insemination), Feet edema, Large varicose veins, Sexual harassment rape, Mood changes, Amenorrhoea, Urinary-genital system symptoms, Vaginal capsules, Triptorelin medicine, Mental condition-related symptoms, Levothyroxine medicine, Blood group type B, Fertilization difficulties, Rubella, Breast lumps, High blood pressure, Number of miscarriages with current partner, Interstitial bleeding, Ear problems, Pelvic inflammatory

disease (PID), Rh+, Incorrect cytological examination, Nadroparin medicine, Exposition to mutagenic compounds, Stool problems, Oral contraceptive pills, Thyroid abnormalities, Thinking disorders, Long healing, Rh-, Numbness, Lung disease, Blood group type A, Sleep disorders, Sensation of smell, Cervical conisation, Head injury, Insulin resistance, Infertility, Spooning, Intrauterine injection, Mononucleosis, Years of trying to get pregnant, Blood group type AB, Bleeding after intercourse, Recurrent vaginitis, Urinary incontinence, Removal of varicose veins (partner), Tonsils, Number of pregnancies, Weakness, Urethritis, Low blood pressure, Anemia, Diagnosed infertility, Egg or semen reception, Number of biological children with current partner, Unexplained vaginal bleeding, Excessive hair, Pains on walking, Gall bladder problems, Breaks and states after fractures, Weak appetite, Abundance of menstruation, More than two miscarriages, No anticonception, Bleeding bruising, Ovarian cysts, Nausea, Metformin medicine, Heat intolerance, Recurrent bleeding nose, Genital herpes, SARS (severe acute respiratory syndrome), Polyps, Teeth problems, Progesterone medicine, Obesity, Dysuria, Chest pain, Persistent rash, Vascular thrombosis, Other psychic abnormalities, Fainting, Increased bleeding time, Feeling generally healthy, Chlamydiosis, Diabetes, Hysteroscopy, Tumor, Disturbing symptoms related to the circulatory system, ET (embryo transfer) or IVF (in vitro fertilization), Sepsi, Vitamin D, Enlarged and sore breasts, Rheumatic diseases, Pelvic pain, Irregular periods, The maximum difference between the shortest and longest periods, Cytomegaly, Neurological diseases, Blood transfusions, Nerve root, Hepatitis, Symptoms related to eyes - ears - nose, Throat system-related symptoms, Nasal congestion, First menarche, Chickenpox, Plaid tails, Autoimmune diseases, Persistent pain in the neck area, Age at menarche, Cabergoline tablets, BMI, Recurrent miscarriages, Spit out of breast nipple, Permanent contraception, Drainage of fallopian tubes, FSH (follicle-stimulating hormone) medicine, Dydrogesterone medicine, Hernia, Dizziness, Digestive system-related symptoms, Lymphatic system-related symptoms]

S2 Appendix. Columns used in the modeling as a result of the combination of each feature selection method [Urinary-genital system symptoms, Hysteroscopy, Recurrent vaginitis, Tonsils, Myomas, Appendectomy, Diagnosed infertility, Pelvic pain, Cytomegaly, Removal of ovarian cysts, Frequent urination, Reduction of sex drive, Cesarean section, Ovarian cysts, Headaches, Cervical conisation, Drainage of fallopian tubes, Spooning, Hernia, Feeling generally healthy, BMI, Patient age, Longest menstrual cycle length, Shortest menstrual cycle length, Average menstrual cycle length, Number of pregnancies, Number of miscarriages, Years of trying to get pregnant, Oral contraceptive pills, Pelvic inflammatory disease (PID), First menarche].
